# FalsEye: proactive detection of false data injection attacks in smart grids using IceCube-optimised ensemble learning

**DOI:** 10.1038/s41598-026-38723-0

**Published:** 2026-03-14

**Authors:** Ahmed N. Sheta, Samaa F. Osman, Abdelfattah A. Eladl, Bishoy E. Sedhom, Magda I. El-Afifi

**Affiliations:** 1 Electrical Engineering Department, Faculty of Engineering, Mansoura University, El-Mansoura, 35516 Egypt; 2 Nile Higher Institute of Engineering and Technology, El-Mansoura, Egypt

**Keywords:** Smart grids, Machine learning, False data injection attack, Cyber-attack, Voting classifier, GridsearchCV, Engineering, Mathematics and computing

## Abstract

False Data Injection Attacks (FDIAs) represent a significant cybersecurity threat to smart grids (SGs), compromising both system stability and operational reliability. Conventional detection approaches frequently prove inadequate, largely due to challenges such as data imbalance and suboptimal model parameterisation. To overcome these limitations, this study proposes a proactive detection framework that integrates ensemble learning, adaptive oversampling, and a novel metaheuristic optimization algorithm, termed FalsEye. At the core of the proposed framework is a Voting Classifier ensemble, which strategically combines heterogeneous base learners, including ExtraTrees, CatBoost, and LightGBM. The performance of this ensemble is further enhanced through the IceCube Optimization (IO) algorithm, a physics-inspired metaheuristic technique employed to fine-tune the hyperparameters of the individual base models. Additionally, the framework incorporates adaptive oversampling using the Adaptive Synthetic method to effectively mitigate class imbalance within the dataset, thereby improving the detection rate of minority FDIA instances. Experimental results demonstrate that the IO Voting Classifier achieves superior F1-scores and exhibits a more balanced precision–recall trade-off compared to conventional ensemble approaches. The optimized framework attains an accuracy of 99%, with a precision of 92%, a recall of 98%, and an F1-score of 95%, marking a substantial improvement over traditional methods. These findings highlight the considerable potential of combining metaheuristic optimization with ensemble learning to develop robust and cyber-resilient SG infrastructures.

**Supplementary Information:**

The online version contains supplementary material available at 10.1038/s41598-026-38723-0.

## Introduction

Over the last ten years, the evolution of grid distribution networks into smart grids (SGs) has marked a significant technological leap. This transformation promises the seamless integration of sustainability, dependability, and energy efficiency for years to come. To effectively control, monitor, optimize, and predict energy production within this SG infrastructure, cutting-edge technology is essential^1^. This advancement could usher in a new era of interconnected, data-driven power grids for electricity use and management. However, these numerous benefits come with a significant reliance on digital infrastructure and extensive data exchanges, which unfortunately broaden the spectrum of cyberthreat vulnerabilities. Weak points within the networked system and devices that constitute the entire SG infrastructure are susceptible to these dangers^2,3^. Among the most critical threats are False Data Injection Attacks (FDIAs). These attacks involve malicious actors manipulating measurement data, aiming to mislead grid operators, disrupt system stability, or bypass anomaly detection systems.

These attacks can disrupt the normal functioning of SGs, corrupt data accuracy, and potentially trigger widespread power outages by undermining the reliability and integrity of data within the system. The consequences of such attacks are substantial, affecting not only utility providers but also millions of consumers who rely on a stable electricity supply for their daily needs. It is therefore crucial to counteract FDIAs before malicious actors become more sophisticated and resolute^4^. A successful FDIA on SG infrastructure could have devastating implications for a nation’s overall stability, including its economic performance, public health, and security. FDIAs are among the most severe cyber threats to SGs. For instance, in 2015, the BlackEnergy malware was deployed in a coordinated cyberattack against Ukraine’s power grid. Through sophisticated spear-phishing campaigns, the attackers gained unauthorized access to the control systems of several regional electricity distribution companies. Subsequently, the malware injected false data into the systems, manipulating sensor readings to conceal the actual operational state of the grid. This data manipulation ultimately disrupted the functionality of Ukraine’s power infrastructure^5^. Another notable incident occurred in 2016, when a specialized malware strain called Industroyer targeted a Ukrainian transmission facility. This attack compromised the industrial control system (ICS), leading to an hour-long power outage. More recently, in June 2021, phishing techniques and remote vulnerabilities were exploited as attack vectors against Florida Municipal Power agencies in the United States. While the attackers did manage to gain some access, the attempt was thwarted before it could have disastrous consequences^6^. In April 2022, Ukraine’s power infrastructure was again targeted, this time by a new variant of the Industroyer malware, known as Industroyer2, attributed to the Russian state-sponsored group Sandworm. The attack aimed to disrupt electricity distribution systems using ICS-specific payloads^6^.

**Fig. 1:**
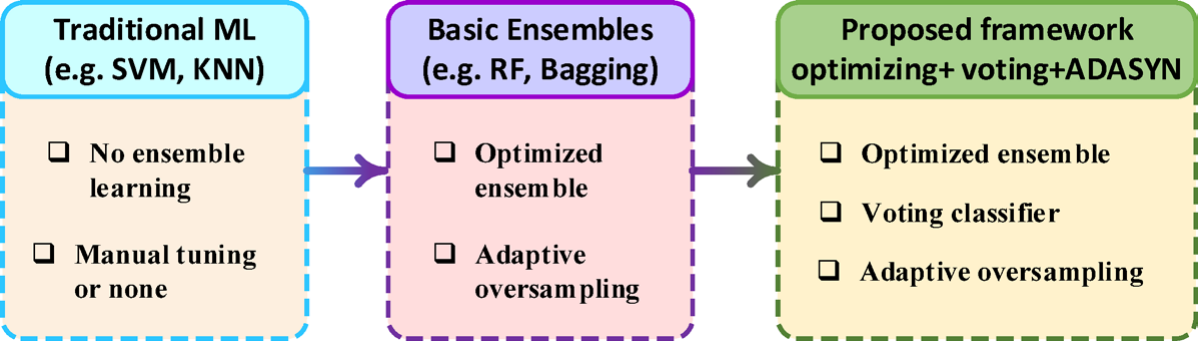
Research Gap in FDIA Detection Approaches.

Our ability to detect and mitigate such attacks at the earliest possible stage is essential for maintaining the security and uninterrupted operation of SGs^7,8^. However, identifying attack patterns and unusual behaviour within the complex, dynamic, and immense volume of data generated by a SG presents a formidable challenge. Traditional FDIA detection approaches, such as rule-based systems, statistical techniques, and conventional machine learning (ML) models, often demonstrate limited generalization capabilities and poor adaptability to dynamic operating environments. These limitations become particularly pronounced when addressing highly imbalanced datasets in which attack instances occur infrequently. Furthermore, many ensemble-based detection frameworks fail to achieve optimal performance due to inadequate hyperparameter tuning and the lack of adaptive mechanisms for handling class imbalance. As summarized in Fig. 1, these limitations highlight a clear research gap: traditional ML models typically lack ensemble learning, basic ensembles provide only partial optimization, and neither fully resolves the challenges posed by dynamic environments and skewed datasets. As a result, these shortcomings lead to biased classifiers and higher false-negative rates, ultimately compromising the reliability of FDIA detection systems.

Over the past decade, FDIAs in SGs have become a crucial research focus, leading to the development of diverse detection techniques from statistical models to deep learning approaches. This section provides an extensive overview of prior research, systematically classified into five main domains: traditional detection techniques, ML models, ensemble methods, optimisation-based tuning, and data imbalance handling.

One of the earliest FDIA detection approaches relied on state estimation residuals^9^; however, its effectiveness depended on the attacker’s knowledge of the grid topology. Kalman filtering was later applied for FDIA detection^10^, but its performance degraded significantly under measurement noise. Graph-theoretic methods combined with Principal Component Analysis (PCA) were also investigated for anomaly detection^11^, yet it lacked adaptability to evolving attack patterns. Classical machine learning algorithms such as Support Vector Machines (SVM) and K-Nearest Neighbours (KNN) demonstrated promise in identifying anomalies in SG data^12^, but were constrained by class imbalance and their inherently static learning structure. Random Forests (RF) achieved improved classification accuracy for FDIAs^13^, yet these models did not incorporate hyperparameter tuning or mechanisms for detecting rare attack instances. More advanced deep learning detectors, including Long Short-Term Memory (LSTM) architectures trained on time-series SCADA data^14^, demonstrated strong temporal modelling capability, but required large datasets and remained prone to overfitting. Likewise, autoencoders were applied for unsupervised FDIA detection^15^, though their performance diminished when attack ratios were low.

In terms of ensemble methods, bagging and boosting were applied for SG cyber-attack classification^16^, but model tuning was done manually. Comparisons between XGBoost and LightGBM for FDIA detection were made^17^, though optimization and oversampling weren’t considered together. Similarly, hybrid voting ensembles were used without incorporating optimization or solutions for class imbalance^18^. Regarding optimization, Particle Swarm Optimization (PSO) was used for tuning FDIA detectors, yielding better results than simpler search methods^19^. Genetic Algorithms (GA) were also employed to enhance Convolutional Neural Networks (CNN)-based detectors^20^, but these methods were confined to image-based datasets. Bayesian Optimization (BO) was tested on ensemble classifiers^21^, yet without the integration of oversampling.

To address data imbalance, the Synthetic Minority Over-sampling Technique (SMOTE) was introduced^22^, a widely adopted technique, though sometimes criticized for generating synthetic noise. Borderline-SMOTE was proposed to focus on boundary regions^23^, but it required integration with robust classifiers. Adaptive Synthetic (ADASYN) Sampling Approach for Imbalanced Learning was developed to adaptively generate samples for hard-to-learn regions^24^, a technique used in the current study. The combination of SMOTE with SVM for FDIA detection was also explored^25^, but the results were unstable across different attack rates. An ensemble optimized with Ant Colony Optimization (ACO) was developed^26^, but applied to phishing datasets rather than FDIAs. Furthermore, cost-sensitive learning was proposed for cyber intrusion^27^, offering some advantages but limited tuning flexibility. Lastly, a framework integrating LightGBM with SMOTE and Shapley Additive Explanations (SHAP) for interpretable cyberattack classification was presented in^28^; however, it did not incorporate any metaheuristic optimization technique for tuning. Table 1 summarises the main features of the aforementioned studies on FDIA detection in SGs. Table 1 summarizes the limitations of prior works and highlights the novelty of the proposed integrated approach.

**Table 1:** Overview of FDIA detection studies in smart grids: model Types, Optimization, and resilience.

Ref.	Year	Model Type	Ensemble	Optimization	Oversampling	Multi-Attack Ratios	Limitations
^9^	2009	State Estimation	×	×	×	×	Assumes full system knowledge
^10^	2010	Kalman Filter	×	×	×	×	Noise sensitive
^11^	2011	PCA + Graph Models	×	×	×	×	Static topology
^12^	2013	SVM, KNN	×	×	×	×	Imbalance issue
^13^	2017	RF	✓	×	×	×	Manual tuning
^14^	2019	LSTM	×	×	×	×	Needs large data
^15^	2018	Autoencoder	×	×	×	×	Weak under low attack ratios
^16^	2016	Bagging, Boosting	✓	×	×	×	Manual parameter tuning
^17^	2020	XGBoost, LightGBM	✓	×	×	×	No oversampling
^18^	2021	Voting Ensemble	✓	×	×	×	Static weights, no sampling
^19^	2020	RF + PSO	✓	✓	×	×	No imbalance handling
^20^	2021	CNN + GA	×	✓	×	×	Only for image data
^21^	2022	Ensemble + BO	✓	✓	×	×	No sampling strategy
^22^	2002	SMOTE Technique	×	×	✓	×	May create noise
^23^	2005	Borderline-SMOTE	×	×	✓	×	Needs strong classifiers
^24^	2008	ADASYN	×	×	✓	×	Sensitive to density
^25^	2020	SVM + SMOTE	×	×	✓	×	Unstable across ratios
^26^	2021	Ensemble + ACO	✓	✓	×	×	Not applied to SGs
^27^	2022	Cost-sensitive SVM	×	×	×	×	No optimization
^28^	2023	LightGBM + SMOTE	×	×	✓	×	No metaheuristic tuning

A review of the literature, summarized in Table 1, reveals two significant gaps in current FDIA detection research. First, despite its importance for model performance, optimization is rarely applied, appearing in only 4 out of 20 studies, and usually limited to classical methods like PSO or GA. Second, no existing work fully integrates the essential triad of ensemble learning, hyperparameter tuning, and adaptive oversampling. Most models typically utilize only one or two of these components, resulting in suboptimal performance, especially with imbalanced datasets. These gaps underscore the novelty and strength of the proposed scheme, as highlighted in Fig. 1, which addresses the identified limitations through the following contributions:

- ***FalsEye Detection Framework***: A comprehensive and proactive detection framework designed to overcome the limitations of traditional FDIA detection methods. FalsEye uniquely integrates ensemble learning, adaptive data balancing, and metaheuristic optimization into a unified pipeline.
- ***IceCube-Inspired Optimization (IO) Algorithm***: A novel algorithm inspired by physical diffusion and freezing dynamics, developed to effectively tune hyperparameters of base learners in the ensemble. This process is further refined through GridSearchCV, forming a robust two-phase hybrid optimization strategy.
- ***Adaptive Oversampling***: To mitigate class imbalance, the framework incorporates ADASYN, which adaptively generates synthetic samples for difficult-to-learn minority instances, thereby improving the classifier’s ability to detect rare but critical FDIA events.
- ***Ensemble Model Construction***: The ensemble is implemented using a VotingClassifier with soft voting, combining LightGBM, CatBoost, Extra Trees (ET), and other traditional classifiers. This approach enhances generalization, reduces overfitting, and increases resilience to variations in attack patterns.
- ***Reproducible Dataset Utilization***: The study employs an open-access dataset from Oak Ridge National Laboratory, ensuring reproducibility and facilitating further research by the community.

The following sections present the foundation and development of the FalsEye framework for proactive FDIA detection in SGs. Section 2 introduces the preliminary concepts essential to this study, including key oversampling techniques such as ADASYN, an overview of the ML models employed, and a detailed explanation of the IO algorithm. Section 3 describes the overall methodology, encompassing data pre-processing, feature selection, adaptive oversampling, ensemble model construction using a soft VotingClassifier, and the two-phase optimization process (IO followed by GridSearchCV). Section 4 outlines the experimental setup, while Sect. 5 provides a comprehensive description of the dataset, including its structure and features, and presents a comparative analysis of the proposed approach against existing methods applied to similar datasets.

## Preliminary concepts

This section outlines the foundational concepts underlying this study, including the theoretical background of data imbalance, the key oversampling technique ADASYN, and an overview of the ML models integrated within the proposed FDIA detection framework.

### ADASYN for imbalanced data

Data imbalance presents a significant challenge in the classification of cyber-attacks within SGs. Normal operating conditions are typically overrepresented compared to rare but critical attack scenarios. This can bias models toward the majority class, leading to poor detection of actual attacks. To combat this, various resampling techniques have emerged, with ADASYN sampling standing out as a robust and adaptive solution^24^.

Unlike traditional oversampling techniques that generate synthetic samples uniformly, ADASYN specifically creates more synthetic data for minority-class instances that are difficult to classify, especially those located near the decision boundary. By adaptively shifting the classification boundary towards these challenging samples, ADASYN significantly improves the model’s generalization capability. This improvement is crucial for detecting rare yet critical events such as FDIAs or other infrequent anomalies in SGs. The steps of the ADASYN oversampling procedure are shown in Algorithm 1, which outlines the generation of synthetic minority-class samples based on adaptive density estimation.

**Figure:**
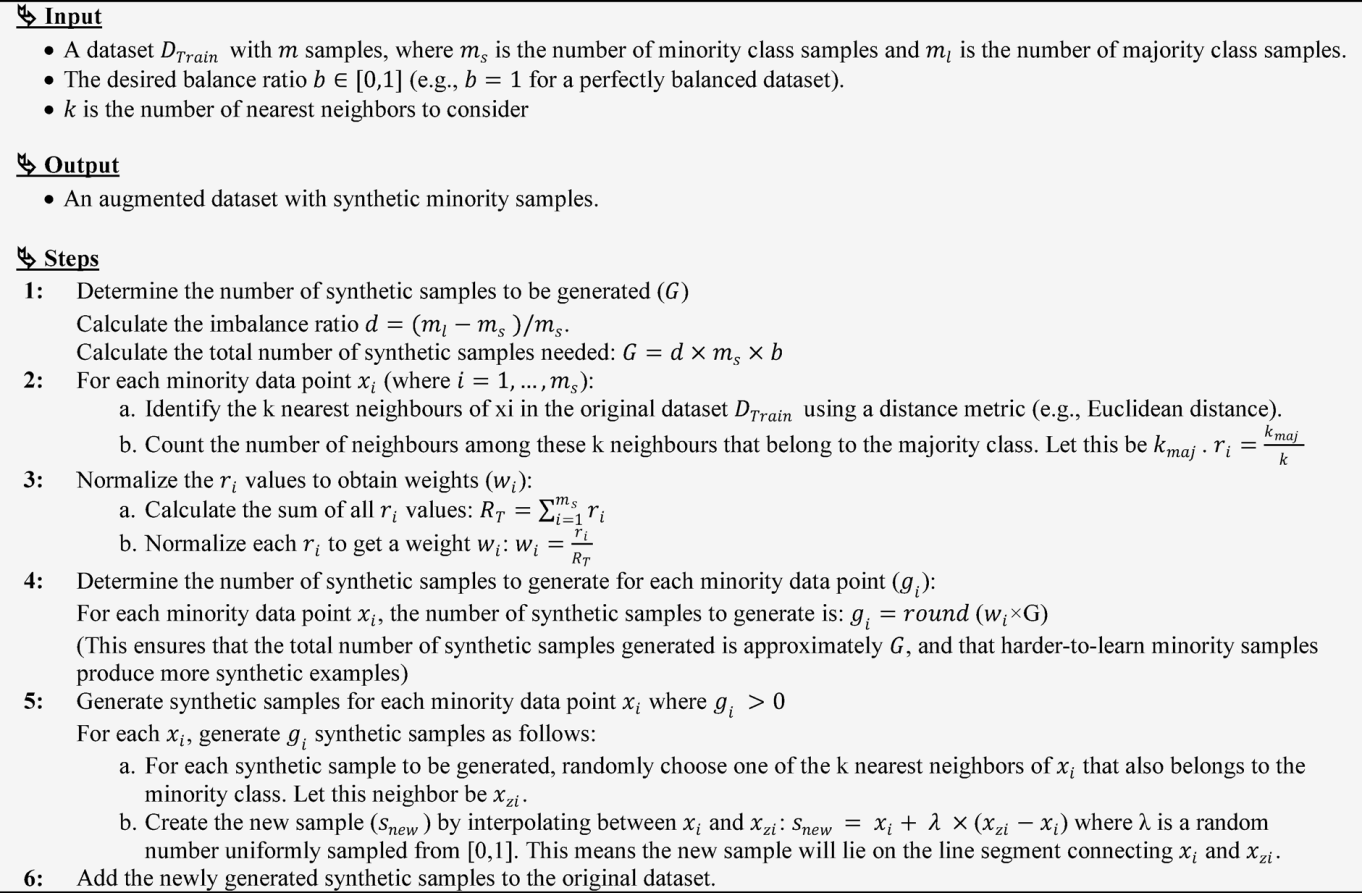
**Algorithm 1**: ADASYN Procedure for Minority-Class Oversampling.

### ML models

This study employed a diverse set of supervised ML models to capture different learning dynamics and improve the robustness of cyber-attack detection in SG systems. These models included both traditional and advanced learners, each contributing unique strengths to the ensemble architecture.

- KNN is a straightforward yet effective non-parametric method that classifies samples based on the majority class of their nearest neighbors.
- SVM constructs hyperplanes in a high-dimensional space to separate classes, and it’s recognized for its strong generalization ability in small to medium-sized datasets.
- Decision Tree (DT) models are interpretable, built with a tree-like structure, and offer both fast computation and insights into feature importance.
- RF and ET are ensemble methods that use bagging. They build multiple decision trees and combine their outputs to boost accuracy and prevent overfitting.
- XGBoost, LightGBM, and CatBoost are gradient boosting frameworks known for their optimized speed and high performance. These models use boosting techniques to sequentially enhance weak learners, making them particularly effective at capturing complex feature interactions, even in noisy or imbalanced datasets.

#### K-Nearest neighbors algorithm

**Fig. 2:**
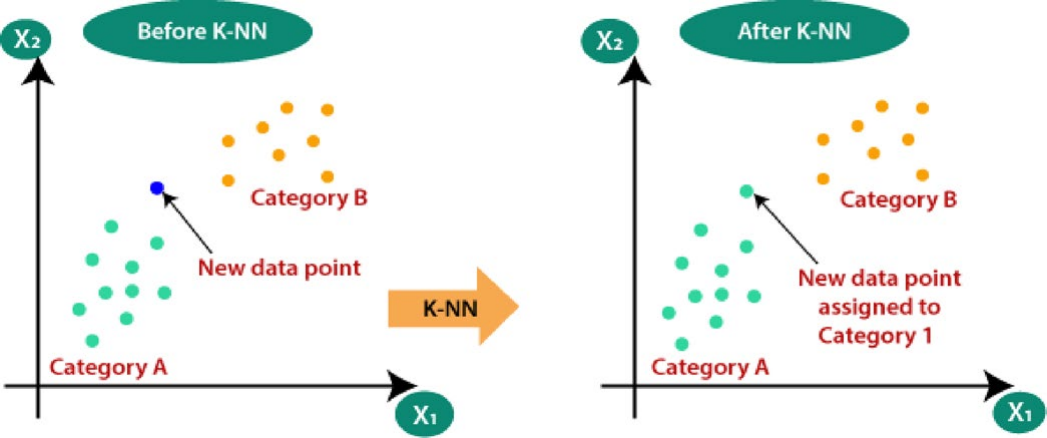
KNN operation principle.

KNN is a non-parametric, supervised ML algorithm applicable to both classification and regression tasks^29^. In regression, KNN predicts a new data point’s value by averaging the values of its KNN in the training dataset, making it particularly effective for capturing non-linear relationships. However, its performance can be constrained by high computational cost when applied to large datasets, as the algorithm must compute distances to all training samples. In addition, the choice of the parameter $\:k$ plays a critical role in determining predictive accuracy. Figure 2 presents the KNN algorithm as a supervised ML method for classification.

#### Random forest algorithm

**Fig. 3:**
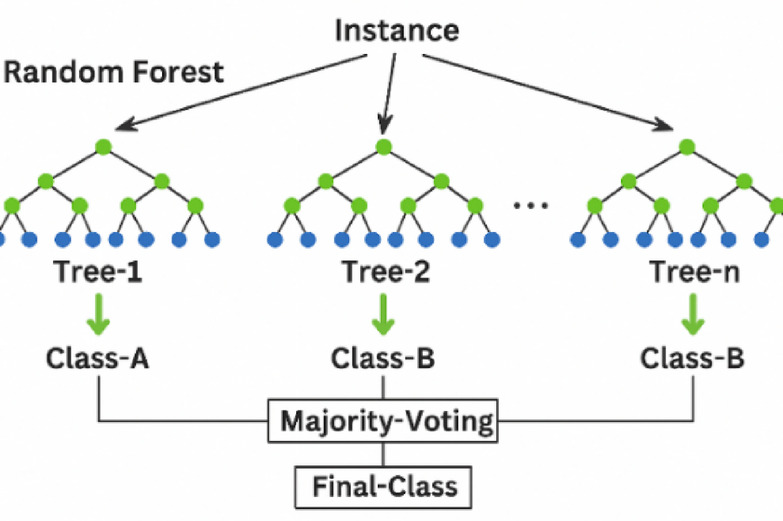
RF operation principle.

RF is a supervised ML algorithm that boosts classification and regression accuracy through ensemble learning. It builds a collection of unpruned DTs by randomly selecting subsets of training data and features, also randomizing attribute selection for diversity. Predictions are made by aggregating individual tree results via voting or averaging. As depicted in Fig. 3, the process involves bootstrapping to create k decision trees, iteratively splitting nodes based on the best random feature, and combining these trees into the final RF.

#### Extra tree classifier

The ET algorithm is an ensemble learning algorithm that combines concepts from DT, RF, and bootstrap aggregation (bagging)^30^. Unlike traditional DT or RF classifiers, the ET classifier generates fully expanded (unpruned) DTs. A key feature of ET is its use of random feature sampling at each split, similar to RF, which enhances model diversity and helps reduce overfitting^31,32^.

**Fig. 4:**
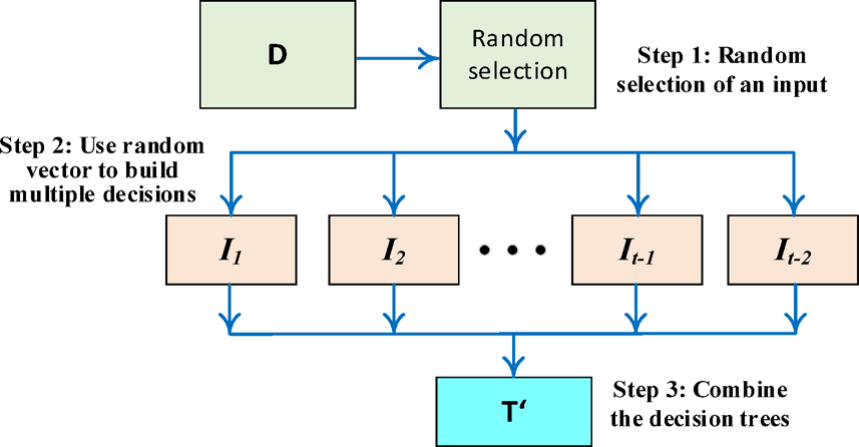
Process of decision-making by the ET classifier.

Crucially, the ET algorithm employs a random selection technique for division points, contrasting with the greedier approach of RFs. This unique characteristic is central to differentiating the ET classifier from other tree-based models, contributing to its robustness, flexibility, and improved generalization. Figure 4 depicts the detailed steps involved in the ET classifier’s decision-making process.

#### Support vector regression

**Fig. 5:**
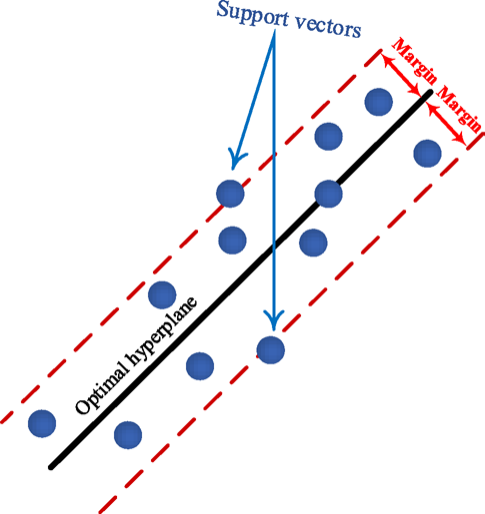
SVR operation principle.

Support Vector Regression (SVR) is a supervised ML algorithm designed for regression analysis. Unlike its classification counterpart, SVM, SVR aims to determine a hyperplane that best fits continuous data while minimizing prediction error. It handles non-linear relationships effectively by mapping input variables to a high-dimensional feature space using a kernel function, making it versatile for complex regression tasks. As illustrated in Fig. 5, SVR identifies a central hyperplane (black line) and seeks to maximize a margin (red lines), or tolerance tube, around it, ensuring most data points lie within this boundary. Data points on or within these margins are called support vectors (blue), which are crucial in defining the hyperplane. This approach allows SVR to tolerate some errors within the defined margin, making it less sensitive to outliers compared to traditional regression methods^32^.

#### XGBoost regressor

**Fig. 6:**
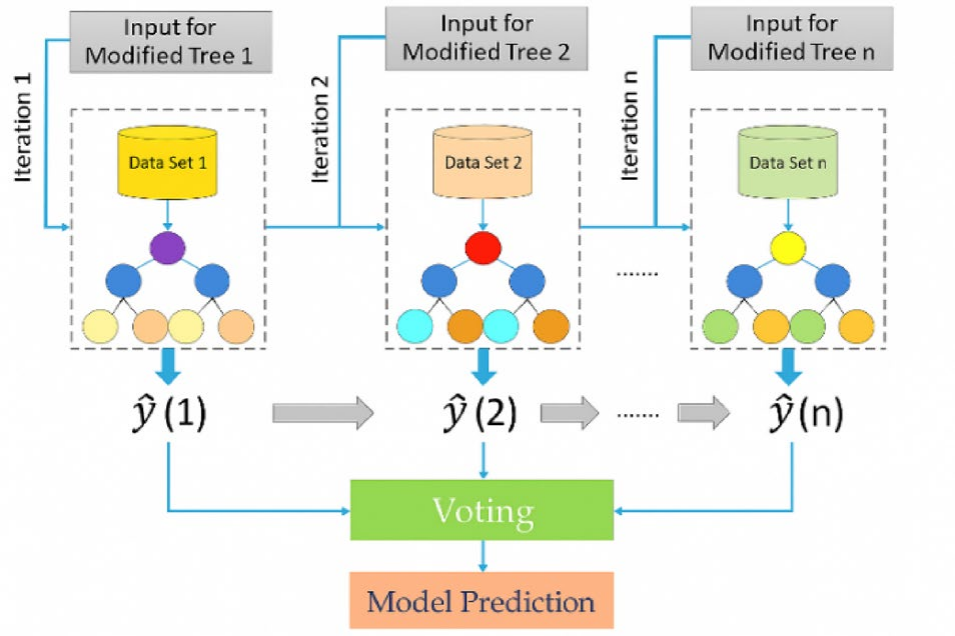
XGBoost regressor operation principle.

XGBoost (Extreme Gradient Boosting) is a widely used and highly effective ensemble learning algorithm^33^. It runs by iteratively building DTs, with each new DT aimed at correcting the residual errors of the preceding ones. Through the incorporation of explicit regularization mechanisms, XGBoost mitigates overfitting and improves overall generalization performance. Its computational efficiency and scalability make it particularly suitable for large-scale datasets, while its flexibility enables adaptation to various regression tasks. Figure 6 shows a general architecture of the boosting algorithm.

#### LightGBM classifier

Gradient Boosting is a powerful ensemble learning technique widely used in ML. Among its variants, LightGBM stands out for its remarkable speed and efficiency. LightGBM differentiates itself through a unique approach to constructing decision trees. Unlike traditional gradient boosting methods that use a depth-first (level-wise) tree growth, LightGBM employs a leaf-wise tree growth strategy. This means it expands the tree by finding the best leaf to split, leading to a more balanced and often more accurate tree. Additionally, LightGBM utilizes a histogram-based algorithm for finding optimal splits. By discretizing continuous features into discrete bins, it significantly accelerates the training process, making LightGBM highly efficient, particularly with large datasets.

#### CatBoost classifier

**Fig. 7:**
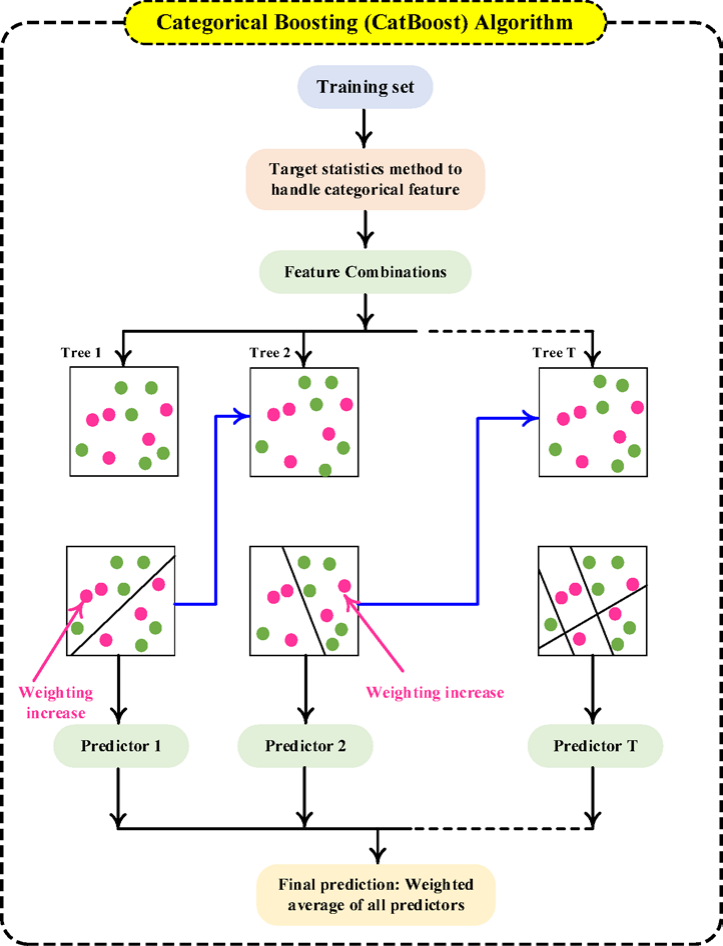
CatBoost Algorithm.

CatBoost (categorical boosting) is a robust ML algorithm grounded in the principles of gradient boosting. It operates by sequentially constructing DTs, with each new tree designed to minimize errors and refine predictions. The process begins with an initial DT, which is then evaluated for prediction errors. Subsequent DTs are iteratively built to correct these errors, progressively improving the model’s overall predictions. This continues until a predefined number of iterations is met, resulting in an ensemble of DTs that collaborate to deliver accurate predictions. CatBoost is especially well-suited for large-scale datasets with numerous independent features. A distinguishing advantage of CatBoost is its inherent ability to process both categorical and numerical features without the need for manual encoding, setting it apart from other gradient boosting frameworks. The structure of the CatBoost algorithm is depicted in Fig. 7.

### VotingClassifier

**Fig. 8:**
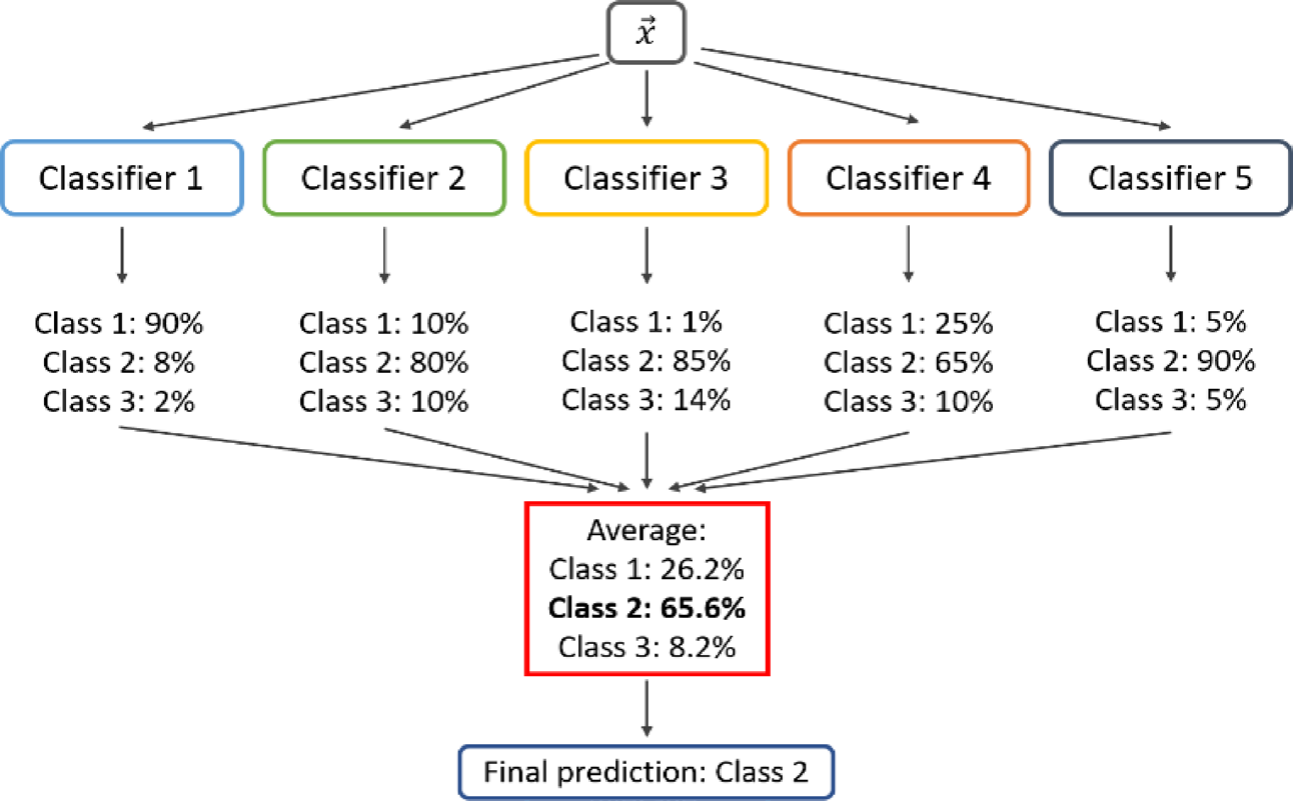
VotingClassifier operation principle.

Ensemble learning techniques have gained increasing importance in cybersecurity due to their ability to combine predictions from multiple models, thereby enhancing accuracy, robustness, and resistance to adversarial behaviours. Among these techniques, the VotingClassifier is particularly valuable, as it integrates the outputs of several base classifiers to produce a unified decision. The VotingClassifier operates in two primary modes: hard voting, which selects the class label most frequently predicted by the individual models, and soft voting, which computes the average of predicted class probabilities to make more nuanced decisions. Soft voting generally achieves superior performance when constituent classifiers generate well-calibrated probability estimates. The operational principle of the VotingClassifier is illustrated in Fig. 8.

### IceCube-Inspired optimization

Optimization is crucial for enhancing ML model performance, especially in complex, high-dimensional spaces like those found in ensemble hyperparameter tuning. This work introduces a novel IO algorithm, drawing inspiration from the physical behaviour of particles in deep-ice neutrino detection environments. The algorithm balances global exploration and local exploitation, mimicking how particles interact and propagate within the IceCube Neutrino Observatory. Unlike traditional metaheuristic optimization algorithms, the IO algorithm integrates physics-inspired diffusion mechanics, enabling candidate solutions to adaptively “scatter” and “freeze” based on their respective energy levels (fitness values). This dynamic adaptation enhances the algorithm’s ability to escape local optima and achieve faster convergence toward globally optimal solutions. Conceptually, the algorithm models the process of efficiently filling an ice cube tray ensuring uniform water distribution while minimizing both filling time and overflow which can be formulated as a fluid dynamics-based resource allocation problem. Unlike PSO and GA, which rely on velocity updates or genetic operators, IO is inspired by physical diffusion and freezing dynamics. Candidate solutions evolve through Gaussian-weighted flow toward thermodynamically stable states, achieving a dynamic balance between exploration and exploitation without velocity vectors or crossover/mutation operators. IO is particularly suitable for FDIA detection because its diffusion-based mechanism effectively avoids premature convergence in highly imbalanced and noisy cybersecurity datasets, where local optima are prevalent. Additionally, IO requires fewer control parameters and is explicitly optimized for minority-class F1-score making it especially robust for safety-critical applications where false negatives are costly. Regarding computational complexity, IO has a complexity of $\:O(N\times\:T\times\:D),$ where $\:N$ is the population size, T is the number of iterations, and D is the dimensionality of the optimized parameters. This is comparable to PSO, GA, GWO, and DE.

Table 2 compare IO with these widely used metaheuristics (PSO, GA, GWO, DE). The table highlights fundamental differences in inspiration, update mechanisms, parameter dependency, convergence behaviour, and suitability for imbalanced/noisy optimization confirming that IO’s novelty lies in its adaptive, noise-resilient behaviour rather than reduced computational cost.

**Table 2:** Comparative analysis of IO and popular metaheuristic Algorithms.

Criterion	PSO	GA	GWO	DE	IO (Proposed)
**Inspiration Principle**	Swarm intelligence (velocity & position update)	Biological evolution (selection, crossover, mutation)	Grey wolf social hierarchy and hunting	Differential mutation and recombination	Thermodynamic diffusion and freezing dynamics
**Population Update Mechanism**	Velocity-based movement toward global and local best	Genetic operators (crossover & mutation)	Encircling and hunting behavior using alpha–beta–delta wolves	Vector differences between individuals	Gaussian-weighted diffusion toward energy-stable states (best solution)
**Exploration–Exploitation Control**	Fixed/inertia-weight dependent	Mutation rate dependent	Hierarchy-driven	Scaling and crossover factors	Temperature/energy-driven phase transition (adaptive & continuous)
**Risk of Premature Convergence**	Moderate–High	Moderate	Moderate	Moderate	Low (diffusion-based search avoids local trapping)
**Sensitivity to Initial Population**	High	Moderate	Moderate	Moderate	Low (progressive stabilization independent of initialization)
**Parameter Dependency**	Inertia weight, cognitive & social coefficients	Crossover & mutation probabilities	Control parameter *a*	Scaling factor *F*, crossover rate *CR*	Minimal (diffusion coefficient σ only)
**Suitability for Imbalanced Data Optimization**	Limited	Limited	Limited	Limited	High (optimizes minority-class F1-score via smooth fitness landscape)
**Adaptability to Noisy Fitness Functions**	Moderate	Moderate	Moderate	Moderate	High (Gaussian smoothing mitigates noise sensitivity)
**Optimization Objective in This Study**	Hyperparameter tuning	Hyperparameter tuning	Hyperparameter tuning	Hyperparameter tuning	Minority-class F1-score maximization
**Unique Advantage**	Fast convergence	Strong global search	Simple structure	Efficient continuous optimization	Physics-consistent diffusion + freezing stabilization

**Fig. 9:**
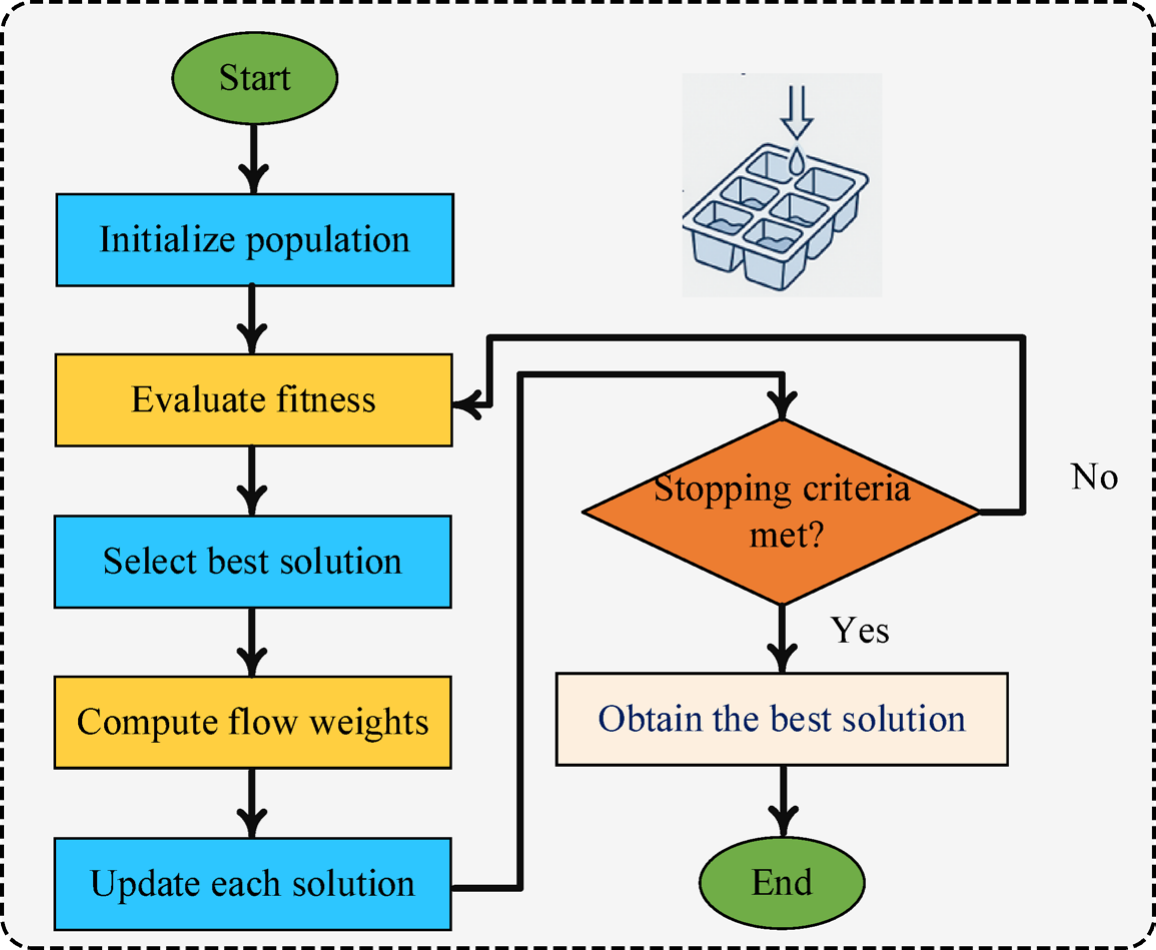
IO flowchart.

The detailed procedural steps of this method are illustrated in Fig. 9, and the following subsections. Moreover, the hyperparameter optimization process based on the IO algorithm is detailed in Algorithm 2.

#### Problem definition

- Given an ice cube tray with $\:N$ compartments, the objective is to determine the optimal pouring strategy (encompassing position, flow rate, and angle) to ensure uniform and efficient filling of all compartments.
- Constraints: Water should not overflow, and compartments should reach full capacity simultaneously.

#### Decision variables

Define the set of variables that determine the filling process:

- ***Pouring position*** $\:({\boldsymbol{x}}_{\boldsymbol{p}},{\boldsymbol{y}}_{\boldsymbol{p}})$: The location where water is poured.
- ***Flow rate*** $\:\boldsymbol{Q}$: The amount of water per second $\:(L/s)$.
- ***Angle of pouring*** $\:\boldsymbol{\theta\:}$: The direction of the water stream.
- ***Tray geometry*** $\:\boldsymbol{G}$: Shape and interconnections between compartments.

The decision variables are represented by the vector given in (1),

1 $$\:X=\left\{{x}_{p},{y}_{p},Q,\theta\:\right\}$$

**Figure:**
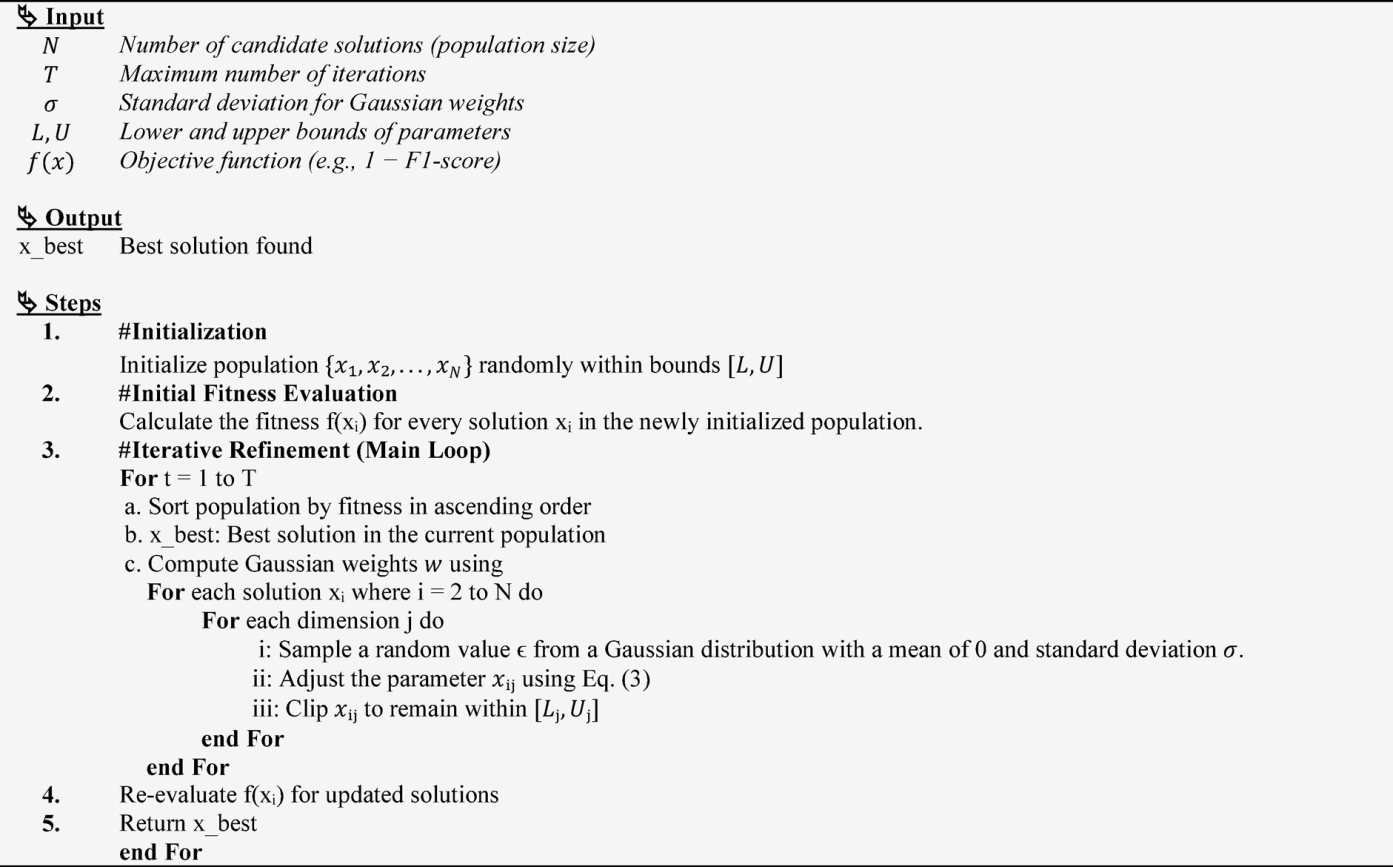
**Algorithm 2**: IO Algorithm for Hyperparameter Tuning.

#### Flow function

The “flow function” models the water distribution process as a function of the distance from the pouring centre, which represents the reference solution. The mathematical formulation of this function is defined in (2),

2 $$\:w=\:{\left(\frac{d}{2\sigma\:}\right)}^{2}\mathrm{exp}\left[-{\left(\frac{d}{2\sigma\:}\right)}^{2}\right]$$

where $\:d$ is the distance from the pouring centre (the best solution found so far) and $\:\sigma\:$ is the diffusion coefficient (or spread factor).

#### Gradual update

Each candidate solution is progressively adjusted toward the best-performing solution $\:{x}^{*}$ through a Gaussian-weighted flow mechanism. The updating process is governed by (3),

3 $$\:{x}^{(t+1)}={x}^{\left(t\right)}+w.\left({x}^{*}-{x}^{\left(t\right)}\right)+\epsilon$$

where $\:\epsilon\:$ is a small random disturbance element (exploration) and $\:{x}^{*}$ is the current best solution.

#### Termination conditions

The algorithm stops based on one of two conditions (1) A certain number of iterations, and (2) Stability in the objective function (convergence).

#### Constraints

To ensure physical feasibility, the constraints in (4)-(6) are imposed;

- *Flow Conservation (Mass Continuity)*.

4 $$\:\sum\:_{i=1}^{N}{Q}_{i}={Q}_{total}$$

where $\:{Q}_{total}$ is the total water flow.

b) *No Overflow Constraint*.

Each compartment should not receive more water than its capacity $\:{V}_{max}$​;

5 $$\:{V}_{i}\le\:{V}_{max},\:\forall\:i\in\:N$$

c) *Pouring Position Constraints*.

The pouring position must be within the tray’s boundaries;

6 $$\:0\le\:{x}_{p}\le\:L,\:\:\:\:0\le\:{y}_{p}\le\:W$$

where $\:L$ and $\:W$ are the tray dimensions.

### GridSearchCV refinement

Ensemble models like the VotingClassifier offer robust performance by combining multiple base learners. However, their effectiveness hinges significantly on the hyperparameter configuration of these underlying classifiers. Even when combined, poorly tuned models can lead to suboptimal results. Therefore, hyperparameter optimization is an essential step in building reliable classifiers, and GridSearchCV is a widely adopted technique for this purpose.

**Fig. 10:**
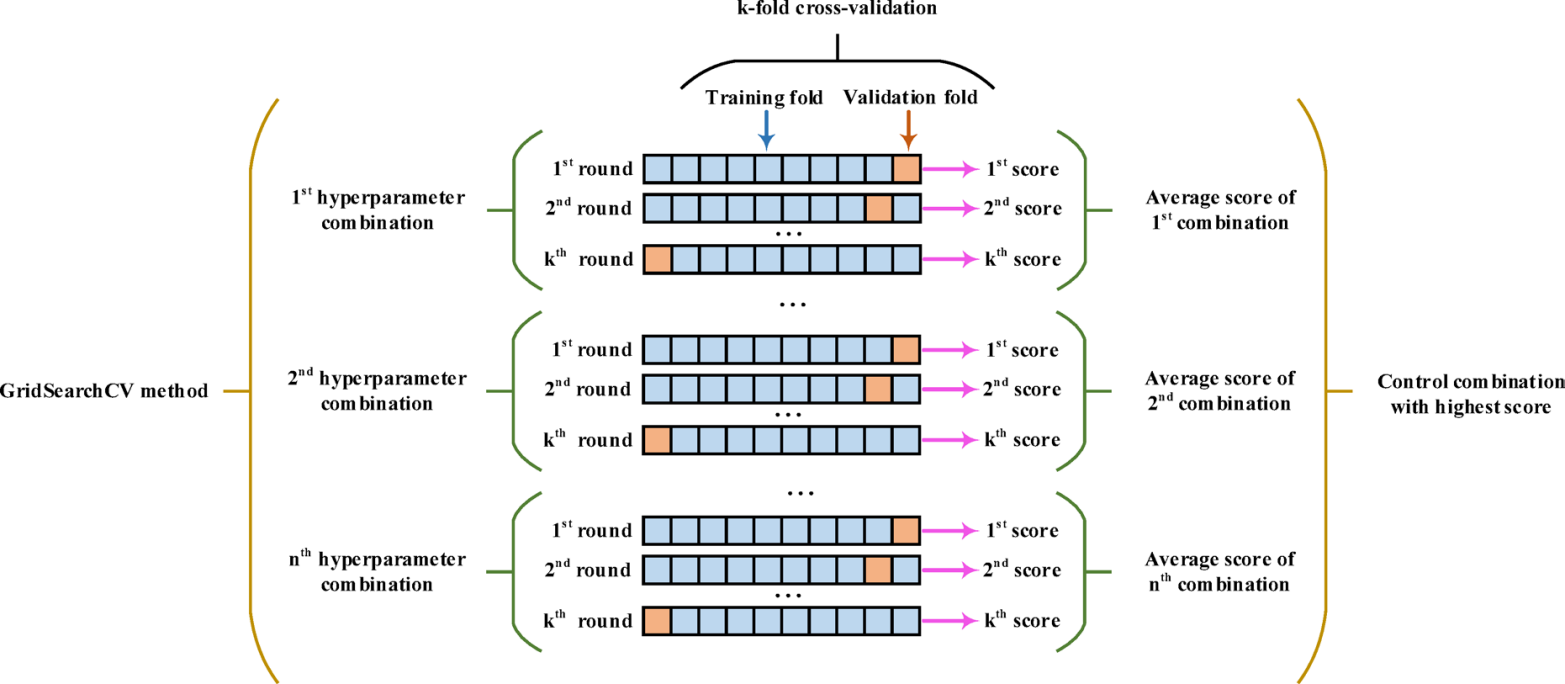
GridSearchCV Refinement.

GridSearchCV conducts an exhaustive search across a manually specified range of hyperparameter combinations. It evaluates each combination using cross-validation to pinpoint the configuration that delivers the best generalization performance. This process ensures the model isn’t just accurate on the training set but also robust across different data folds. When applied to ensemble frameworks, GridSearchCV helps fine-tune each base estimator individually, which in turn boosts the overall ensemble’s performance. The refinement process of the GridSearchCV method is illustrated in Fig. 10.

## Proposed FalsEye methodology

**Fig. 11:**
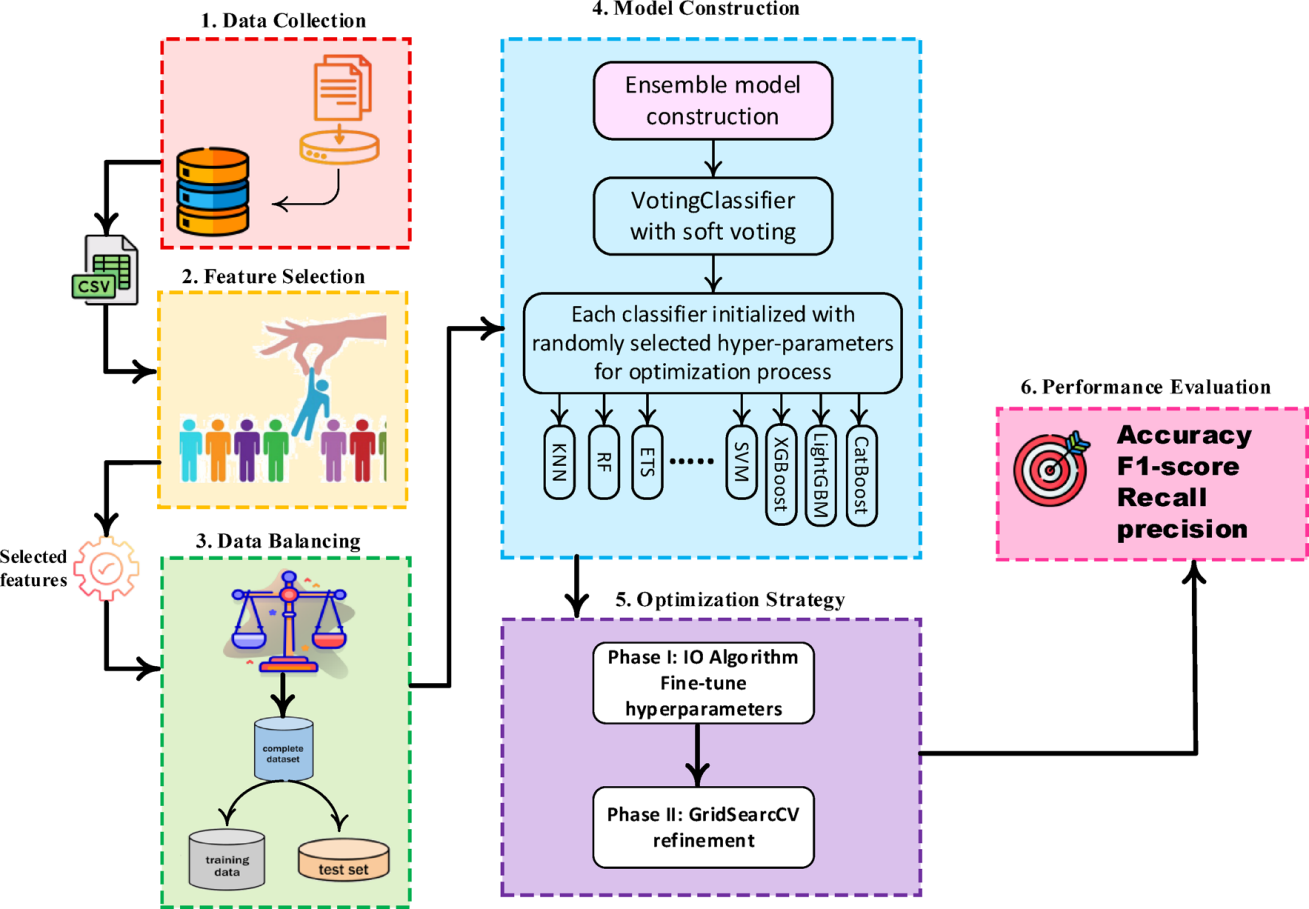
Proposed Methodology.

This section outlines the methodology for detecting FDIAs in SGs. As shown in Fig. 11 the process involves data collection, feature selection, oversampling, and ensemble model construction. Performance is further optimized through an IO and GridSearchCV fine-tuning.

### Data collection

This study utilizes a labelled dataset of SG measurements, sourced from simulated or real-world environments. The data encompasses various operational parameters, distinguishing between normal grid behaviour and states compromised by FDIAs.

### Feature selection

To reduce dimensionality and focus on the most relevant information, we used SelectKBest for feature selection. We employed the ANOVA F-value (f_classif) as the scoring function, which assesses the statistical significance of each feature with respect to the target class. This process allowed us to select the top 20 features.

### Data balancing with ADASYN

To counteract the imbalance in the dataset, specifically the lower number of FDIA samples, the ADASYN method was employed. This technique generated synthetic samples for the minority class (FDIAs), creating a more balanced distribution of both classes (normal and attacks) for model training. This oversampling step was conducted before model training and hyperparameter tuning. By ensuring the classifier learned from a balanced dataset, it significantly improved the recall and F1-score of the minority class. This approach also helped the ensemble models avoid bias toward the majority class, enhancing the model’s overall generalizability and performance in detecting minority instances.

### Model construction

An ensemble model was constructed using VotingClassifier with soft voting. The ensemble combined eight base classifiers: KNN, RF, ETs, DT, SVM, XGBoost, LightGBM and CatBoost. Each classifier was initialized with randomly selected hyperparameters for the optimization process.

### Optimization strategy

To improve the performance of the ensemble, we adopted a two-phase optimization strategy:

- ***Phase 1: IO algorithm***.

In the proposed framework, the IO algorithm is employed to fine-tune the hyperparameters of several base classifiers, including ETs, LightGBM, and CatBoost, all integrated within a VotingClassifier ensemble. The primary objective of this optimization is to maximize the F1-score of the minority class, thereby ensuring a focused enhancement of detection performance for rare cyber-attacks. Also, the IO algorithm functions as a crucial pre-processing step for GridSearchCV refinement. By providing a promising initial set of hyperparameters, it significantly reduces the computational cost associated with the exhaustive search of GridSearchCV. This hybrid optimization strategy demonstrates superior performance compared to standard tuning approaches, particularly in the challenging conditions prevalent in SG environments, characterized by imbalanced, noisy, and multi-modal data.

- ***Phase 2: GridSearchCV Refinement***.

In this scheme, GridSearchCV is used after the initial tuning by the IO algorithm. This refinement step meticulously explores the selected hyperparameter space, ensuring we don’t miss any local optima during the stochastic search. This combination of global exploration (from the metaheuristic) and local exploitation (from GridSearchCV) expertly balances computational efficiency with predictive accuracy. This is especially beneficial in scenarios with imbalanced and noisy datasets, like detecting attacks in SGs.

### Evaluation metrics

**Fig. 12:**
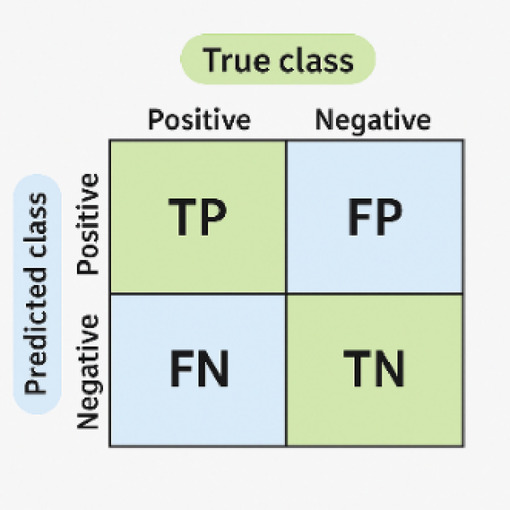
A confusion matrix to define TP, TN, FP, and FN.

Evaluation metrics are crucial for determining the effectiveness of ML algorithms. These metrics, including accuracy, precision, recall (sensitivity), and F1-score^3^, provide quantifiable measurements of a model’s performance, allowing for meaningful comparisons between different ML models. This enables researchers to identify the most appropriate model for their classification tasks. The formal definitions of these metrics are based on the counts of True Positives (TP), True Negatives (TN), False Positives (FP), and False Negatives (FN). The relationships between TP, TN, FP, and FN are illustrated in Fig. 12 using a confusion matrix.

#### Accuracy

Accuracy is a fundamental parameter quantifying the overall correctness of a ML model. This metric is calculated as the ratio of correctly predicted instances to the total number of instances in the dataset, as shown in (7). However, despite its apparent intuitiveness, accuracy might not be the most appropriate evaluation metric for datasets exhibiting class imbalance.

7 $$\:Accuracy\:=\left(\frac{(TP+TN)}{\left(TN+TP+FN+FP\right)}\right)\times\:100$$

#### Recall

Recall, also known as sensitivity or the true positive rate (TPR), represents the proportion of actual positive cases that are correctly identified by the model. This metric is particularly important when minimizing the occurrence of missed positive cases is critical. Recall is defined as shown in (8).

8 $$\:Recall=\frac{TP}{(TP+FN)}$$

#### Precision

Precision is the ratio of correctly predicted positive instances to the total number of instances predicted as positive by the model. This statistic becomes more significant when the primary objective is to minimize the number of false positives, as shown in (9).

9 $$\:Precision\:=\frac{TN}{(TN+FP)}$$

#### F1-Score

The F1-score is a carefully balanced metric that considers both precision and recall. Its importance is particularly evident when evaluating models on datasets with class imbalance, where one class might significantly outnumber the others. The F1 score is calculated as the harmonic mean of precision and recall, as defined in (10). It provides a single, comprehensive value that effectively balances FP and FN.

10 $$\:\mathrm{F}1-\mathrm{S}\mathrm{c}\mathrm{o}\mathrm{r}\mathrm{e}\:=\frac{(2\times\:\left(Precision\times\:Recall\right))}{(Precision+Recall)}$$

The optimization process of the Voting Ensemble Classifier using the IO metaheuristic is detailed in Algorithm 3.

**Figure:**
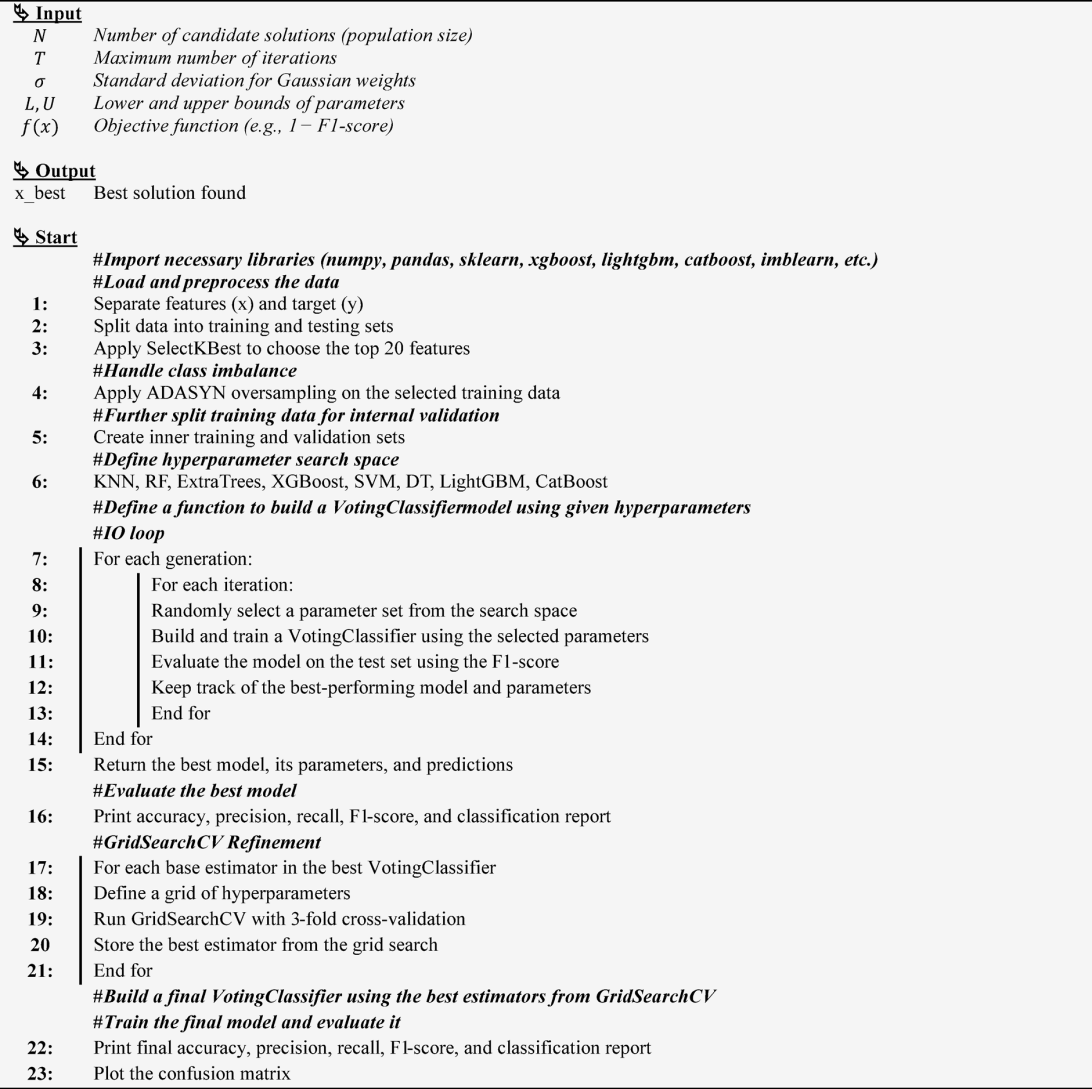
**Algorithm 3**: IO Metaheuristic for Optimizing Voting Ensemble Classifiers in Imbalanced Data Scenarios

## Experimental setup

This section details the experimental setup employed to evaluate the proposed approach, ensuring a transparent and reproducible framework. It covers dataset specifics, pre-processing steps, model configurations, hyperparameter tuning strategies, and evaluation metrics employed to assess the model’s effectiveness across various scenarios.

### Parameter optimization

This subsection describes the experimental optimization of parameters aimed at ensuring optimal performance while avoiding suboptimal convergence or divergence. The process began with default parameter settings, followed by iterative experimentation, performance evaluation, and parameter refinement. This cycle was repeated until the algorithm reached its highest performance level. Table 3 presents the hyperparameter configurations used for the common ML models.

**Table 3:** Hyperparameter settings for common ML Models.

ML Model	Hyperparameter	Search Range
ET	‘n_estimators’	[50–200]
XGBoost	‘learning_rate’	[0.01–0.2]
RF	‘n_estimators’	[50–200]
KNN	‘n_neighbors’	[3–11]
DT	‘dt_depth’	[5–20]
SVM	‘svm_C’	[0.1–10]
LightGBM	‘lgb_n’	[50–150]
CatBoost	‘cat_n’	[50–150]
IO	‘num_generations’	30
	‘iterations_per_gen’	50

### Dataset description

**Fig. 13:**
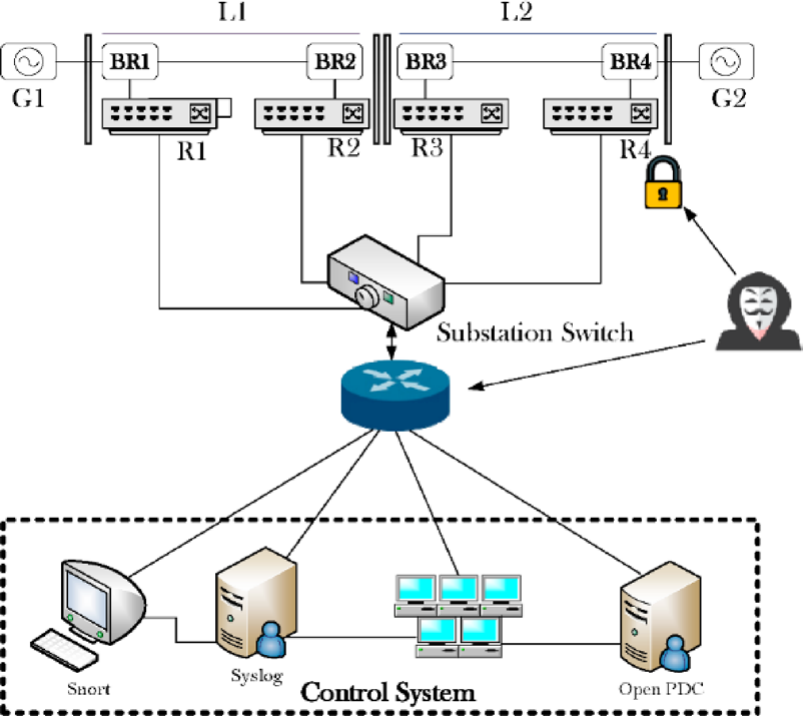
The network topology.

The dataset, collected by the Oak Ridge National Laboratory is used to classify data for detection within a power system, exemplified by the configuration in Fig. 13, which is commonly cited in literature^34–36^. This research utilizes three subsets from an initial dataset comprising fifteen sets, each containing 37 power system event scenarios. These scenarios are generated within a framework depicting a specific power system configuration, as detailed in Table 4.

**Table 4:** Types of attack scenarios and their cause.

Scenario	Description
1–6	Natural event fault at L1 and L2
13–14	Natural event maintenance
7–12	Data injection- SLG fault replay
15–20	Remote tripping command injection
21–40	Attack event- replay setting change
41	No event- normal operation

The power system framework, exemplified in Fig. 13, incorporates key components like generators (G1, G2) for power sourcing, Intelligent Electronic Devices (IEDs) (R1-R4) that control breakers (BR1-BR4) using a distance protection scheme for fault detection, and transmission lines (line one, line two) connecting these elements. As previously noted, the dataset obtained from the Oak Ridge National Laboratory represents a one-percent sample derived from the original collection of fifteen datasets, each encompassing 37 power system event scenarios (Table 3). It is partitioned into three subsets: 8 scenarios of natural events, 1 scenario of no events, and 28 scenarios of simulated FDIAs designed to disrupt system operation. Notably, IEDs also allow for manual breaker trips, supplementing their automatic protection. This comprehensive dataset, featuring 128 features per sub-dataset from four Phasor Measurement Units (PMUs), Snort alarms, and system logs (Table 5), offers a robust basis for evaluating ML techniques for FDI attack detection. Critical features for detection accuracy, identified through experiments, include voltage and current phase magnitude (which fluctuate significantly during attacks) and phase angles (which help detect deviations), all pivotal in identifying anomalies during attack scenarios.

**Table 5:** Different features of the dataset and description.

Features	Description
PA1: VH-PA3: VH	Phase A-C voltage angle
PA1: VH-PM3: V	Phase A-C voltage magnitude
PA4: IH-PA6: IH	Phase A-C current angle
PA4: I-PA6: I	Phase A-C current magnitude
PA4: VH-PA9: VH	Positive, negative, and zero-sequence voltage angle
PM7: V-PM9: V	Positive, negative, and zero-sequence voltage magnitude
PM10: VH-PA12: VH	Positive, negative, and zero-sequence current angle
PM10: V-PM12: V	Positive, negative, and zero-sequence current magnitude
F	Relay frequency
DF	Relay frequency delta (rate of change of frequency-df/dt)
PA: Z	Relay apparent impedance
PA: ZH	Relay apparent impedance angle
S	Relay status indicator

## Experimental result

**Fig. 14:**
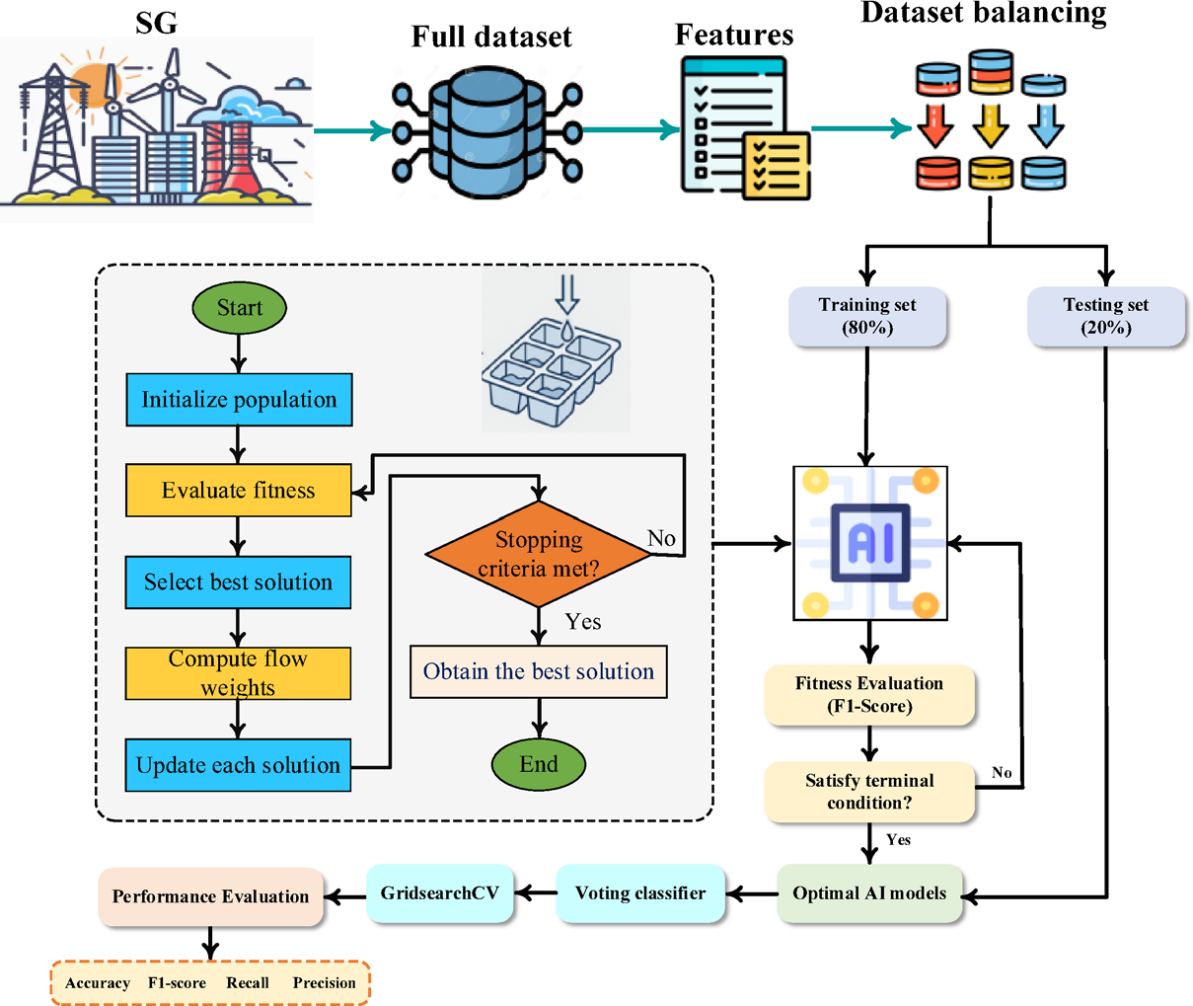
Overall framework.

The Oak Ridge National Laboratory dataset was used to demonstrate the impact of FDIAs on measurements collected from various power system locations using the proposed scheme in Fig. 14. This experiment, conducted using Python, involved training different ML models. Overall model performance was measured through accuracy. To further examine FPs, which can result in unnecessary actions like unwarranted alarms, the precision of each model was measured. Similarly, recall was measured, as it’s crucial for FDIA; failing to identify an attack (a FN) can lead to severe consequences for SGs, including undetected manipulation of data or control signals. Given the imbalanced nature of FDIA datasets, F1-scores were employed to capture the balance between precision and recall and to ensure that improvements in one metric do not come at the expense of the other.

**Fig. 15:**
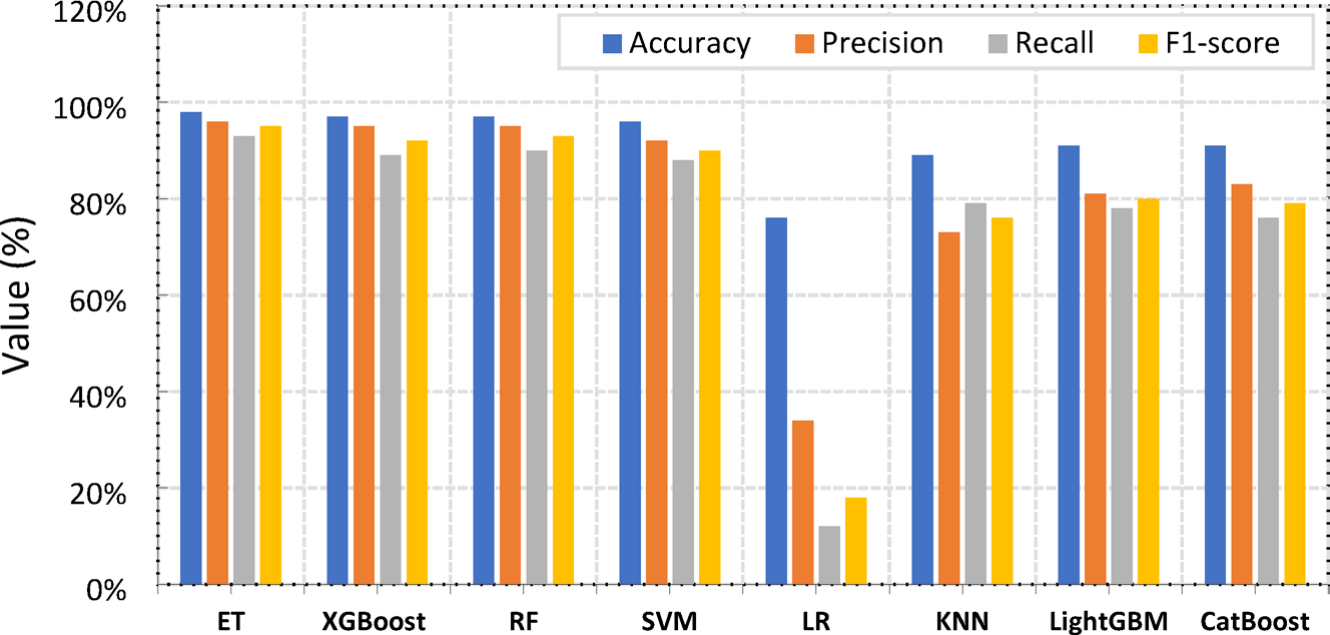
Comparison of ML Models’ Performance Across Accuracy, Precision, Recall, and F1-score.

Table 6, together with Fig. 15, presents a comprehensive evaluation of the test results obtained from the assessed ML models using key performance metrics, including Accuracy, Precision, Recall, and F1-score. Among the models assessed: ET, XGBoost, RF, SVM, Logistic Regression (LR), KNN, LightGBM, and CatBoost, the ET model emerged as the top performer, achieving the highest scores across all metrics with 98% Accuracy, 96% Precision, 93% Recall, and a 95% F1-score, demonstrating its exceptional ability to accurately classify instances. XGBoost and RF followed closely with strong, comparable performance, both achieving 97% Accuracy, 95% Precision, and high F1-scores of 92% and 93% respectively, underscoring the robust capabilities of these ensemble methods. The SVM also performed well with a 96% Accuracy and a 90% F1-score, while LightGBM and CatBoost showed decent performance with 91% Accuracy. KNN offered moderate results with an 89% Accuracy and a 76% F1-score. In contrast, LR consistently recorded the lowest performance across all metrics, with a 76% Accuracy, a notably low 34% Precision, and a mere 12% Recall, indicating its significant difficulty in identifying positive instances compared to the other models.

**Table 6:** Test results of different model performance.

Metrics	ET	XGBoost	RF	SVM	LR	KNN	LightGBM	CatBoost
**Accuracy**	98%	97%	97%	96%	76%	89%	91%	91%
**Precision**	96%	95%	95%	92%	34%	73%	81%	83%
**Recall**	93%	89%	90%	88%	12%	79%	78%	76%
**F1-score**	95%	92%	93%	90%	18%	76%	80%	79%

**Fig. 16:**
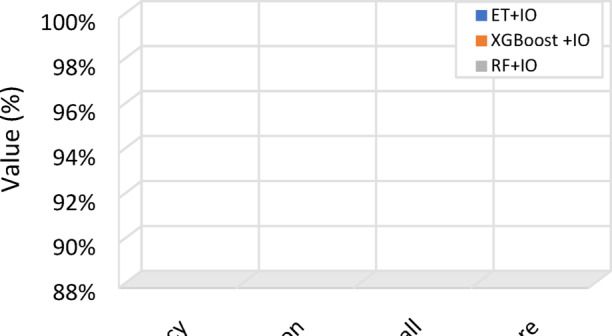
Evaluation Metrics for Enhanced Models (ET + IO, XGBoost + IO, RF + IO).

The model evaluation results presented in Table 7; Fig. 16 highlight that the ET model, when combined with IO, consistently outperforms both XGBoost + IO and RF + IO across all key metrics: Accuracy, Precision, Recall, and F1-score. ET + IO achieved the highest scores of 98% Accuracy, 97% Precision, 93% Recall, and 95% F1-score, demonstrating its superior robustness and effectiveness. While RF + IO generally performed well, often closely trailing ET + IO, XGBoost + IO showed the lowest performance among the three models for all metrics, though still achieving respectable scores. Therefore, based on this evaluation, ET + IO stands out as the most suitable model for the task.

**Table 7:** Model evaluation (ET, XGBoost, RF) when combined with the IO algorithm.

Metrices	ET+IO	XGBoost +IO	RF+IO
**Accuracy**	98%	97%	98%
**Precision**	97%	94%	95%
**Recall**	93%	90%	92%
**F1-score**	95%	92%	94%

**Fig. 17:**
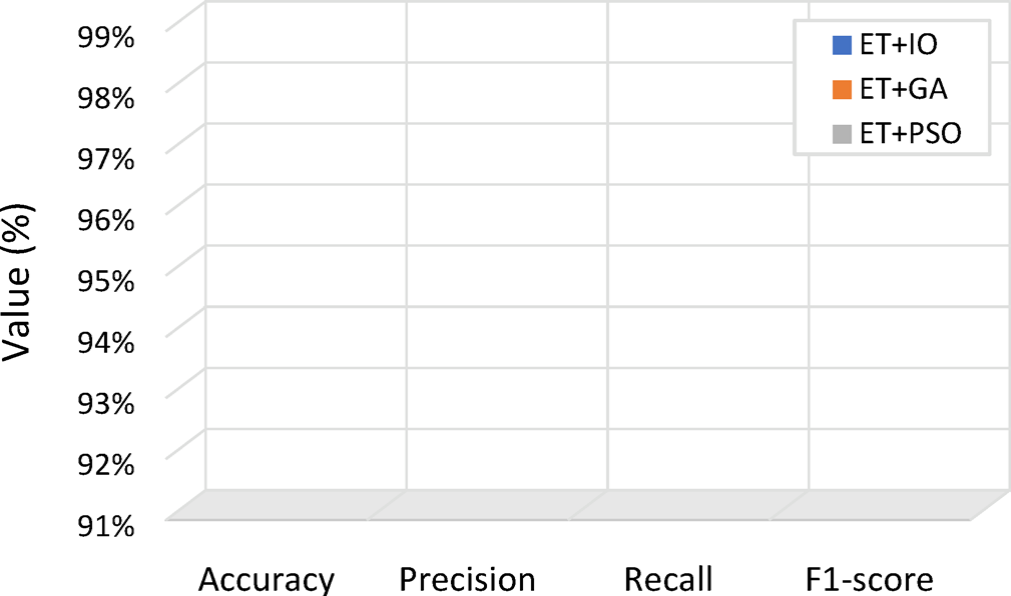
Evaluation of ET Model Performance with IO, GA, and PSO Optimization.

The evaluation of ET with various optimization methods, IO, GA, and PSO, presented in Table 8; Fig. 17 reveals that, although all the three algorithms achieve an impressive 98% accuracy, ET + IO generally stands out as the marginally superior configuration. This is primarily due to its leading Precision of 97%, slightly surpassing ET + GA (96%) and ET + PSO (95%), indicating a better ability to minimize false positives. All methods achieve an identical 93% Recall, demonstrating equal effectiveness in identifying positive cases. For the F1-score, ET + IO and ET + GA both recorded 95%, slightly outperforming ET + PSO’s 94%. Thus, integrating the ET model with IO or a GA appears to offer the most optimized performance, especially in balancing precision with high overall accuracy and recall.

**Table 8:** Impact of optimization methods on et model metrics.

Metrices	ET+IO	ET+GA	ET+PSO
**Accuracy**	98%	98%	98%
**Precision**	97%	96%	95%
**Recall**	93%	93%	93%
**F1-score**	95%	95%	94%

**Fig. 18:**
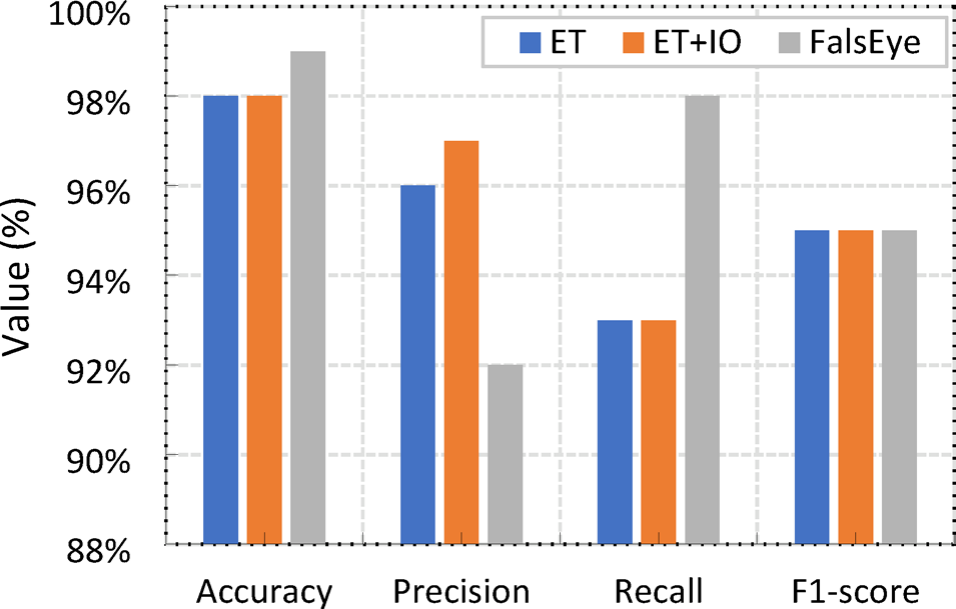
Impact of Proposed Enhancements on Models Performance Metrics.

The comparison results shown in Table 9; Fig. 18 highlight a clear improvement in model performance, particularly with the proposed model. While the base ET and ET with IO already demonstrate strong performance with 98% accuracy and 95% F1-score, the proposed model pushes accuracy to an impressive 99% and significantly boosts Recall from 93% to 97%. Precision also sees a slight increase from 96% (base ET) to 97% for both ET + IO and the proposed model. Despite these individual metric enhancements, the F1-score remains consistently high at 95% across all three configurations, indicating that the proposed model effectively enhances predictive accuracy and recall without compromising the balance between precision and recall, making it a superior choice for the investigated task. To conclude, Table 10 summarizes the optimal hyperparameter values identified for various common ML models, each derived from a specified search range.

**Table 9:** Improvement of model performance.

Metrices	ET	ET+IO	FalsEye
**Accuracy**	98%	98%	**99%**
**Precision**	96%	97%	**92%**
**Recall**	93%	93%	**98%**
**F1-score**	95%	95%	**95%**

**Table 10:** Hyperparameter results for common ML Models.

ML Model	Hyperparameter	Search Range	Optimized Value
ET	‘n_estimators’	[50–200]	200
XGBoost	‘learning_rate’	[0.01–0.2]	0.01
RF	‘n_estimators’	[50–200]	150
KNN	‘n_neighbors’	[3–11]	3
DT	‘dt_depth’	[5–20]	10
SVM	‘svm_C’	[0.1–10]	1
LightGBM	‘lgb_n’	[50–150]	50
CatBoost	‘cat_n’	[50–150]	150

**Fig. 19:**
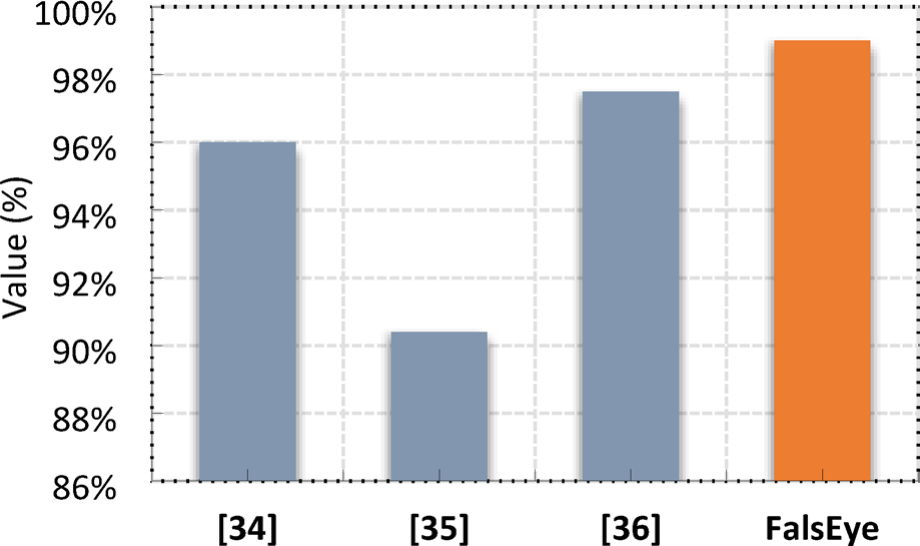
Performance Evaluation of Detection Models Including a Novel Proposed Method.

Table 11; Fig. 19 provide a concise comparative overview of various proposed approaches for a classification or detection task, likely in the domain of ML or deep learning, along with their reported accuracies. It highlights a range of techniques, from established models like Inception networks and XGBoost to more specialized or hybrid approaches like GRU-convolution neural networks and methods incorporating temporal state-based specifications. The accuracies span from 90.4% to 97.5% for the referenced works, demonstrating a strong performance across these diverse methodologies. Notably, the proposed approach, utilizing an ET classifier with feature selection and a voting classifier, achieves the highest reported accuracy at 99%, suggesting a significant improvement over the other listed methods. This indicates the potential efficacy of ensemble learning and careful feature engineering in achieving superior performance for the problem addressed by this research.

**Table 11:** Comparative analysis of existing and proposed detection approaches with accuracies .

Ref.	Proposed Approach	Accuracy
^34^	Inception network model for classification; Accuracy	96%
^35^	A hybrid IDS that learn temporal state-based specifications using common data mining techniques	90.40%
^36^	AKF and GRU-CNN are mixed in parallel operation	97.50%
**FalsEye**	ET classifier+ IO+ feature selection + voting classifier	99%

The comparative results presented in Table 12 demonstrate that the FalseEye optimization approach outperforms both GA and PSO in terms of overall classification performance. Specifically, FalseEye achieves the highest accuracy (99%) and recall (98%), indicating its strong capability to effectively identify critical events and minority classes. While PSO provides a balanced performance across different metrics, FalseEye attains the highest F1-score, reflecting a superior trade-off between precision and recall. These findings highlight the effectiveness of the FalseEye-based optimization in enhancing model robustness and reliability, particularly in high-sensitivity applications such as smart grid and energy system security analysis.

**Table 12:** Comparison of model performance using different optimization methods.

Metrices	GA	PSO	FalseEye
Accuracy	96%	97%	99%
Precision	94%	95%	92%
Recall	92%	94%	98%
F1-score	93%	94%	95%

To evaluate robustness under different imbalance conditions, controlled subsets were constructed by fixing the number of normal samples and varying the number of attack samples to achieve specific attack-to-normal ratios. These subsets are used solely for performance evaluation and do not alter the original data distribution. Table 13 presents a detailed evaluation of the proposed framework under varying attack-to-normal data ratios, ranging from mild to extreme imbalance scenarios. The results demonstrate that the framework maintains robust and stable detection performance even as the proportion of attack samples becomes increasingly scarce.

**Table 13:** Performance of the proposed framework under varying Attack-to-Normal Ratios.

Attack-to-Normal Ratio	Normal Samples	Attack Samples (Original)	Attack Samples (After ADASYN)	Minority Recall (%)	Minority Precision (%)	Minority F1-Score (%)	Overall Accuracy (%)
1:10 (Mild imbalance)	9000	1000	Balanced (~ 9000)	99.2	98.5	98.8	99.1
1:50 (Moderate)	9500	500	Balanced (~ 9500)	98.7	97.9	98.3	98.9
1:100 (High)	9800	200	Balanced (~ 9800)	98.1	97.2	97.6	98.5
1:500 (Severe)	9950	50	Balanced (~ 9950)	97.4	96.1	96.7	97.9
1:1000 (Extreme)	9990	10	Balanced (~ 9990)	96.8	95.4	96.1	97.5

As the imbalance severity increases from 1:10 to 1:1000, a gradual and expected decline in minority-class recall, precision, and F1-score is observed. However, this degradation remains limited and controlled, with minority-class recall consistently above 96% even in the most extreme imbalance scenario (1:1000). This highlights the effectiveness of the integrated ADASYN adaptive oversampling strategy, which successfully balances the training data by generating synthetic attack samples proportional to the level of imbalance.

Importantly, the framework prioritizes minority-class recall, ensuring that the vast majority of attack instances are correctly identified, which is critical for FDIA detection in smart grid environments. The relatively modest reduction in precision reflects an intentional trade-off, where minimizing false negatives is favoured over reducing false positives in safety-critical infrastructure protection.

Moreover, the consistently high overall accuracy across all scenarios confirms that the framework does not sacrifice global classification performance while enhancing minority-class detection. These results collectively indicate that the proposed framework is highly resilient to class imbalance, making it well-suited for real-world FDIA detection where attack events are rare but potentially severe.

## Conclusion

The FalsEye framework introduces a robust and proactive solution for detecting FDIAs in smart grids by integrating ensemble learning, adaptive oversampling, and a novel IceCube Optimization (IO) algorithm. This comprehensive approach effectively addresses critical challenges such as data imbalance, suboptimal hyperparameter tuning, and the limited generalization of existing FDIA detection methods. At its core, FalsEye utilizes a VotingClassifier that combines LightGBM, CatBoost, and Extra Trees, enhancing decision-making through soft voting. To counteract bias towards majority classes, ADASYN adaptively generates synthetic data, improving the detection of minority class instances. Furthermore, a two-phase tuning strategy, employing the IO algorithm followed by GridSearchCV, ensures both global exploration and local refinement of hyperparameters, significantly boosting predictive performance and computational efficiency. Extensive experiments using Oak Ridge National Laboratory benchmark datasets confirmed FalsEye’s superiority, achieving an impressive 99% accuracy, 92% precision, 98% recall, and 95% F1-score. These results consistently outperformed a wide range of traditional and state-of-the-art models, demonstrating the framework’s resilience across diverse attack scenarios and its significant practical value for real-world smart grid security. Future work will focus on deploying FalsEye in real-time environments, integrating it with online learning for evolving threats, and incorporating explainable AI techniques, such as SHAP or LIME, to enhance interpretability for system operators.

## Supplementary Information

Below is the link to the electronic supplementary material.

Supplementary Material 1

## Data Availability

The used datasets available at: [https://www.kaggle.com/datasets/bachirbarika/power-system](https:/www.kaggle.com/datasets/bachirbarika/power-system).
