# Supplementary material for: FalsEye: proactive detection of false data injection attacks in smart grids using IceCube-optimised ensemble learning
Source: Sci Rep. 2026 Mar 14;16:9093. doi: 10.1038/s41598-026-38723-0 (PMC12993076; doi:10.1038/s41598-026-38723-0)
Supplement: Supplementary file 1 — Supplementary Material 1 [file 41598_2026_38723_MOESM1_ESM.docx]

# **Appendix**

To ensure full reproducibility, all experimental configurations, preprocessing steps, optimization parameters, and software specifications are summarized in Tables A1-A4.

Table A1

Data Preprocessing Configuration

| **Item** | **Description** |
| --- | --- |
| Missing value handling | Median imputation (numerical features) |
| Feature scaling | Min-Max normalization to [0,1] |
| Class balancing method | ADASYN |
| ADASYN sampling strategy | auto |
| ADASYN neighbors | 5 |
| Train–test split | Stratified (80% / 20%) |

Table A2

Ensemble Model Composition

| **Component** | **Description** |
| --- | --- |
| Ensemble type | Soft voting |
| Base learners | Random Forest, Extra Trees, XGBoost, LightGBM, CatBoost |
| Voting weights | Equal weights |

Table A3

IceCube Optimization (IO) Parameters

| **Parameter** | **Value** |
| --- | --- |
| Population size | 50 |
| Maximum iterations | 30 |
| Diffusion coefficient | Adaptive: 1.0 → 0.01 |
| Fitness function | Minority-class F1-score |
| Optimization phase | Global hyperparameter search |

Table A4

Fine-Tuning, Software, and Reproducibility Settings

| **Item** | **Specification** |
| --- | --- |
| Local refinement | GridSearchCV |
| Cross-validation | 5-fold stratified |
| Python version | 3.10.12 |
| scikit-learn | 1.3.2 |
| xgboost | 2.0.3 |
| lightgbm | 4.1.0 |
| catboost | 1.2.5 |
| numpy | 1.24.3 |
| pandas | 2.1.4 |
| imbalanced-learn | 0.11.0 |
| Random seed | 42 |
| Seed control | Data split, ADASYN, IO initialization, model training |
